# Combination of urinary biomarkers can predict cardiac surgery-associated acute kidney injury: a systematic review and meta-analysis

**DOI:** 10.1186/s13613-025-01459-7

**Published:** 2025-03-29

**Authors:** Nikolett Kiss, Márton Papp, Caner Turan, Tamás Kói, Krisztina Madách, Péter Hegyi, László Zubek, Zsolt Molnár

**Affiliations:** 1 Centre for Translational Medicine, Semmelweis University, 78 Üllői Str., Budapest, 1082 Hungary; 2 Heart and Vascular Centre, Semmelweis University, Budapest, Hungary; 3 Department of Anaesthesiology and Intensive Therapy, Semmelweis University, Budapest, Hungary; 4 Department of Anaesthesiology and Intensive Therapy, Saint John’s Hospital, Budapest, Hungary; 5 Department of Stochastics, Budapest University of Technology and Economics, Budapest, Hungary; 6 Institute for Translational Medicine, Medical School, University of Pécs, Pécs, Hungary; 7 Institute of Pancreatic Diseases, Semmelweis University, Budapest, Hungary; 8 Poznan University of Medical Sciences, Poznan, Poland

**Keywords:** Cardiac surgery-associated acute kidney injury, Urinary biomarkers, Accuracy, Predictive value, Intraoperative, Postoperative

## Abstract

**Introduction:**

Acute kidney injury (AKI) develops in 20–50% of patients undergoing cardiac surgery (CS). We aimed to assess the predictive value of urinary biomarkers (UBs) for predicting CS-associated AKI. We also aimed to investigate the accuracy of the combination of UB measurements and their incorporation in predictive models to guide physicians in identifying patients developing CS-associated AKI.

**Methods:**

All clinical studies reporting on the diagnostic accuracy of individual or combined UBs were eligible for inclusion. We searched three databases (MEDLINE, EMBASE, and CENTRAL) without any filters or restrictions on the 11th of November, 2022 and reperformed our search on the 3rd of November 2024. Random and mixed effects models were used for meta-analysis. The main effect measure was the area under the Receiver Operating Characteristics curve (AUC). Our primary outcome was the predictive values of each individual UB at different time point measurements to identify patients developing acute kidney injury (KDIGO). As a secondary outcome, we calculated the performance of combinations of UBs and clinical models enhanced by UBs.

**Results:**

We screened 13,908 records and included 95 articles (both randomised and non-randomised studies) in the analysis. The predictive value of UBs measured in the intraoperative and early postoperative period was at maximum acceptable, with the highest AUCs of 0.74 [95% CI 0.68, 0.81], 0.73 [0.65, 0.82] and 0.74 [0.72, 0.77] for predicting severe CS-AKI, respectively. To predict all stages of CS-AKI, UBs measured in the intraoperative and early postoperative period yielded AUCs of 0.75 [0.67, 0.82] and 0.73 [0.54, 0.92]. To identify all and severe cases of acute kidney injury, combinations of UB measurements had AUCs of 0.82 [0.75, 0.88] and 0.85 [0.79, 0.91], respectively.

**Conclusion:**

The combination of urinary biomarkers measurements leads to good accuracy.

**Supplementary Information:**

The online version contains supplementary material available at 10.1186/s13613-025-01459-7.

## Background

Cardiac surgery-associated acute kidney injury (CS-AKI) is a common postoperative complication with an incidence of 20–50%, resulting in increased in-hospital morbidity, mortality and healthcare costs [1, 2]. The diagnosis of acute kidney injury (AKI) is based on increased serum creatinine (sCr), decreased urine output, or both, defined by the KDIGO criteria [3]. However, an increase in sCr occurs only after more than 50% of the glomerular filtration rate (GFR) is lost, which indicates its limited sensitivity in detecting early kidney damage [4].

Renal stress, early damage, and functional urinary biomarkers (UBs) have been extensively investigated to identify patients at high risk of developing CS-AKI and highlight the group of patients who may benefit from specific therapeutic measures. Extensive research on urinary neutrophil gelatinase-associated lipocalin (NGAL), liver-type fatty acid binding protein (L-FABP), interleukin-18 (IL-18), kidney injury molecule (KIM-1), hepcidin-25, N-Acetyl-β-D-glucosaminidase (NAG), tissue-inhibitor of metalloproteinase-2 and insulin growth factor binding protein-7 (TIMP2xIGFBP7), α- and π-glutathione-S-transferase (α-GST, π-GST), α−1-microglobulin, etc. show varying predictive accuracy.

A multi-centre randomised clinical trial reported a significantly reduced incidence of CS-AKI with the introduction of KDIGO-bundle in the early postoperative period in high-risk patients identified by TIMP2xIGFBP7 [5]. Recent expert consensus statements by the Society of Cardiovascular Anaesthesiologists and practice updates by the Acute Disease Quality Initiative (ADQI) group and the ERAS Society Guidelines for Cardiac Surgery recommend the incorporation of urinary biomarkers in the early diagnosis and prevention of acute kidney injury and also cardiac surgery-related acute kidney injury [6–8]. However, the predictive value of individual biomarkers and the potential benefits of combining different biomarkers remain unclear.

Therefore, we aimed to review the available literature to obtain summative information on the predictive accuracy of various urinary biomarkers in CS-AKI to guide clinicians on which urinary biomarker to incorporate into their clinical practice to prevent the development of CS-AKI.

## Methods

**Fig. 1:**
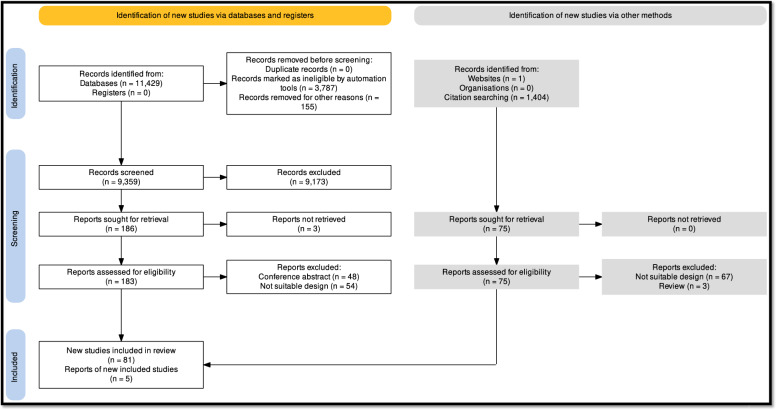
PRISMA 2020 flowchart representing the study selection process

We report our systematic review and meta-analysis based on the recommendation of the PRISMA 2020 guideline (Fig. 1), while we followed the Cochrane Handbook [9, 10]. The study protocol was registered on PROSPERO (registration number CRD42022371166). The PRISMA checklist is available in the Supplementary material.

### Eligibility criteria

All studies reporting on the diagnostic accuracy of urinary biomarkers following cardiac surgery were included in our analysis if they provided AUC-ROC, sensitivity and specificity for predicting cardiac surgery-related acute kidney injury, as defined by the Kidney Disease Improving Global Outcomes (KDIGO) criteria [3]. We did not use any filters regarding specific urinary biomarkers investigations.

### Information sources

Our initial systematic search was conducted in three databases: EMBASE, PUBMED and CENTRAL on the 11th of November 2022. We updated the literature search on the 3rd of November 2024.

### Search strategy

During the systematic search, the following search key was used:

(((urinary) OR (excretion) OR (renal) OR (kidney) OR (urine) OR (stress) OR (damage)) AND (biomarker)) AND ((((cardiac) AND (surgery)) OR (aortic AND surgery) OR (aortic AND valve AND surgery) OR (off-pump) OR ((on cardiopulmonary) AND (bypass)) OR (mitral AND surgery) OR (mitral AND valve AND surgery) OR (coronary AND surgery) OR (on-pump))).

### Selection process

The selection was performed by two independent review authors (NK and MP) using reference management software (EndNote 20, Clarivate Analytics). After manual and automatic duplicate removal, title and abstract selection was followed by full-text selection. Cohen's Kappa was calculated for inter-reviewer agreement at each phase, and progression to the next stage happened if Cohen’s Kappa was greater than 0.80. Disagreements were resolved by consensus and involving a third reviewer (CT).

### Data collection process

All data from all eligible studies were collected in parallel by two independent investigators (NK and MP), in accordance with a predefined data collection plan. Any disagreements between the investigators were resolved by consensus.

### Data items

The following data were extracted from all eligible studies: first author, year of publication, study population, study period, country of origin, number of centres, number of patients included, age and sex of population, preoperative serum creatinine, inclusion and exclusion criteria, types of cardiac surgery, use of cardiopulmonary bypass (CPB), type of urinary biomarker, measurement time points, test manufacturer, biomarker cut-off values and units, CS-AKI definition criteria, CS-AKI time frame, number of patients without and with CS-AKI in terms of stages, the purpose of diagnostic study, sensitivity and specificity with 95% confidence intervals (95% CI), negative predictive value (NPV) and positive predictive value (PPV), area under the receiver operator characteristic curve (AUC-ROC) with 95% CI, standard error (SE) and p-value, true positive (TP), false positive (FP), true negative (TN), false negative (FN), positive likelihood ratio (+ LR), and negative likelihood ratio (−LR).

### Study risk of bias assessment

Two authors (NK and MP) independently performed the risk of bias assessment using QUADAS-2 [11]^.^and GRADE Pro [12] to assess the quality of evidence, with disagreements resolved by another author (CT).

### Synthesis methods

Statistical analyses were carried out using the R statistical software (version 4.1.2.) and the R script of the online tool [13]. A p-value of less than 0.05 was considered significant for all statistical analyses.

We collected AUC values and their confidence intervals. We estimated the standard deviations of the AUC values from the confidence intervals. If no confidence interval was available, we used the published formula [14].

The main statistical challenge is that the AUC values of a study corresponding to different biomarkers typically belong to the same population. To create statistically rigorous pooled AUC values, we had to consider both the correlations between the sample errors and the correlations between the random effects corresponding to the outcomes in the same study. To account for these correlations, we fitted multivariate mixed-effects models using the RMA.mv function of the metaphor R package. We used a hierarchical random effect correlation structure. To circumvent the problem caused by unknown correlations between sample errors, we supplemented the method with the robust approach of Pustejovsky et al. implemented in the coef_test function of the clubSandwhich R package [15]. As an initial guess, we assumed the same correlation between any two miRNA AUC corresponding to the same sample and then applied the robust correction of the clubSandwhich package. We visualised the results in forest plots.

For the meta-analysis of sensitivity and specificity, two by two contingency tables were also extracted/calculated from the studies, which included true positive, false positive, false negative, and true negative values, as well as the corresponding threshold, if available.

In the area of diagnostic meta-analysis, one approach is to calculate summary ROC curve, for example by following Rutter and Gatsonis et al. [16]. An alternative is the bivariate model, which focuses on pooled sensitivity and specificity [17, 18]. Interestingly, it has been shown in the study by Harbord et al. that the two approaches are mathematically equivalent, only the focuses are different [19]. We had enough data only for TIMP2xIGFBP7 for diagnostic meta-analysis. For three specific thresholds, we performed a meta-analysis with the corresponding data, focusing on pooled sensitivity and specificity. We plotted the sensitivity and specificity of the included studies, summary estimates of sensitivity and specificity, and the corresponding 95% confidence and prediction regions on the ROC plot. In these visualisations, the sizes of the ellipsoids reflect the weights of the studies calculated according to the method described previously [20]. The size of the prediction region provides insight into this heterogeneity.

Due to the high heterogeneity and the complications caused by the correlations between the outcomes, we did not perform publication bias analysis.

Biomarker measurement time points varied between studies, as shown in Supplementary Table 1. We merged time points based on the pathophysiology of acute kidney injury and pooled data accordingly. Preoperative, at anaesthetic induction, at the time of Foley catheter placement, time points were merged as ‘preoperative’. All intraoperative measurements and those performed up to 6 h postoperatively were pooled as ‘intraoperative and early postoperative’. We had sufficient data to perform a separate analysis for TIMP2xIGFBP7 for intraoperative measurements and early postoperative measurements with insignificant differences. We therefore decided to keep the merged pooling as ‘intra- and early postoperative’. We merged all measurement time points from 6 to 24 h postoperatively, postoperative days 1, 2 and 3, and performed subgroup analyses accordingly. We pooled ‘early maximum’ measurements separately.

The AUC value is within the range [0.5–1.0], where the minimum value represents the performance of a random classifier and the maximum value corresponds to a perfect classifier (e.g., with a classification error rate equivalent to zero) [14, 21]. In general, an AUC of 0.5 indicates no discrimination (i.e., the ability to diagnose patients with and without the disease or condition based on the test), 0.7 to 0.8 is considered acceptable, 0.8 to 0.9 is considered good, and a value higher than 0.9 is considered excellent [22].

### Outcomes

Our primary outcome was pooled AUC of individual biomarkers for predicting all stages of CS-AKI as defined by the KDIGO criteria. Our secondary outcomes were pooled AUC of individual biomarkers for predicting CS-AKI as defined by the AKIN and RIFLE criteria, pooled AUC of individual biomarkers for severe CS-AKI, and pooled sensitivities and specificities of urinary biomarkers for predicting all and severe CS-AKI [23, 24].

### Protocol deviations

We deviated from our protocol by not only creating Forest plots based on our original registration but also by including additional secondary outcomes and studies in our meta-analysis where the outcome definition of acute kidney injury was based on the AKIN or RIFLE criteria and by pooling and analysing AUC-ROC of a combination of urinary biomarkers for predicting postoperative CS-AKI [23, 24]. Pre-planned subgroup analyses were not performed due to a lack of sufficient data in the literature, such as analyses of the predictive value of urinary biomarkers in patients with co-morbidities that pose a higher risk of CS-AKI. High-risk patient groups were not defined homogeneously among studies; therefore, subgroup analysis was not possible. We performed subgroup analyses based on merged UB measurement time points to statistically handle the between-study heterogeneity regarding measurement times. We qualitatively assessed the length of hospital stay, ICU stay, and 28-day mortality because of the high overall between-study heterogeneity and low-quality evidence.

## Results

### Search and selection

Altogether, during the initial and the updated systematic search, 13,908 studies were identified using our search key, and 11,788 articles were screened by title and abstract following duplicate removal. Two hundred sixty-six studies were found eligible for full-text selection. After excluding 168, we identified 98 eligible articles. 95 articles were included in this review (Fig. 1).

We analysed 87 studies quantitatively; 8 studies were reviewed qualitatively. We found four studies reported in multiple articles that included different biomarker analyses or different outcomes (Supplement Ref 1, 2,3,4,5,6,7,8,9,10).

We pooled data describing the performance of 11 urinary biomarkers: TIMP2xIGFBP7, NGAL, NAG, KIM-1, L-FABP, IL-18, Cystatin-C, α-GST, π-GST, hepcidin-25, and α−1-microglobulin. Data describing hemojuvelin, angiotensinogen, neprilysin, microRNA, ATF3 and trace elements were analysed qualitatively as we had less than three poolable articles for each.

### Basic characteristics of included studies

The baseline characteristics of the enrolled analyses of a total of 14,455 patients are detailed in Table 1. In our studied population, the incidence of CS-AKI was 28.77% (4,159/14,455).

**Table 1:** Basic characteristics of studies included

Author, year, country	Type of study	Number of patients/female %	Age whole/without AKI/with AKI	Type of surgery	Inclusion criteria	Exclusion criteria
Cummings et al. 2019, USA	Prospective cohort of RCT	400/32.7	65 ± 12	CABG, valve, asc aorta, CABG + Valve	Adult, elective CABG, valve, asc aorta surgery	Statin intolerance, ACS, liver dysfunction, CYP3A4 inhibitors, cyclosporine, RRT, RTx, urgent/emergent surgery, pregnancy
Lakhal et al. 2021, France	Prospective observational	65/53	79 (77, 81)	Aortic valve	> 75y, aortic valve on CPB	RRT, additional surg procedure
Zaouter, 2018, France	Prospective observational	50/44	72 ± 8	Valve or combined	Pts high risk for AKI: 2of these: > 60y, severe Llarteriopathy, > 51%carotid stenosis, diabetes, valvular or combined surgery	Dementia, previous sternotomy, offpump, endocarditis, chr RF (GFR < 90), renal artery stenosis, pregnancy, emergent surgery
Finge, 2017, France	Prospective observational	93/26	68 ± 10	CABG, valve, asc aorta	Adult, on CPB, elective and non-elective coron, valve, asc aorta	RRT, pregnancy
Grieshaber, 2019, Germany	Prospective observational	133	65 ± 9	CABG, nonCABG, combined	Adult, elective and non-elective, on CPB, high risk for AKI (LS > 25%, CCS > 6)	RRT
Couturier, 2021, France	Retrospective observational	185/29	66 ± 11	CABG, valve, combined, on pump	Elective on pump surgery	Pregnancy, KTx, CKD (< 30), RRT, VAD, specific AKI (vasc, glomerular, obstructive, intersititial)
Wetz, 2015, Germany	Prospective observational	42/31	72 (65,76)	CABG	Elective and nonelective CABG on CPB	Valve surgery, vasc noncoron surgery, no CPB, HTx, ECMO, RRT
Vandenberghe, 2022, Netherlands	Prospective observational	100/28	68 (58,76)	All elective	Elective on pump, < 90y	Pregnancy, breast feeding, KTx, CKD (< 20), RRT, VAD, AKI, glomerulonephr, interst neph
Esmeijer, 2021, Netherlands	Prospective observational	344/35	66 ± 11	Nd	Elective cardiac	Pregnancy, active infection, emergent surgery
Pilarczyk, 2015, Germany	Prospective observational	60/20	69 ± 9//76 ± 4 (AKI 0–1//AKI 2–3)	CABG onpump	Elective on pump CABG first time	RRT
Piedrafita, 2022, France	Prospective observational	661/24	66 ± 12	CABG, valve, combined, asc aorta,ASD,VSD, Htx	On pump	Unscheduled CPB, RRT
Alam, 2022, USA	Prospective observational	73/20		LVAD, HTx	LVAD, HTx	RRT, multiorgan transplant
Engelman, 2021, USA	Retrospective observational	412/24	70 ± 8	On pump	All cardiac surg, SeCreat < 177, electand non elect, Rtx	RRT
Meersch, 2014, Germany	Prospective observational	50/34	71 ± 12	On pump	All cardiac surg on CPB, CCS > 6	Rtx, pregnancy, immunosuppression, steroid th changed in last 2 weeks
Yimei Wang, 2017, China	Prospective observational	57/28	60 (49, 65)	CABG, valve, valvuloplasty	All cardiac surgery	RRT, RTx
Mayer, 2017, Switzerland	Prospective observational	110		On pump	Elective on pump	RRT, pregnancy, emergent s
Oezkur, 2017, Germany	Prospective observational	150/28	67	CABG, valve, combined, asc aorta	Elective on pump, CABG, valve, combined asc aorta	eGFR < 60, active infection, COMT inhibitor, MAO inhibitor, immunosuppr, pregnancy, lactation
Irqsusi, 2022, Germany	Observational pilot	50/0	68 (58,74)	CABG, valve	Elective, male, CABG, valve, on pump	CS > 7, < 35y, acute infection, emergency, ESRF
Yu, 2022, USA	Prospective observational	108/25	67 (59,73)	Isolated CABG, reop, not isolated CABG	On pump cardiaic surgery	Emergent sy, HTx, VAD, preop assist device, GFR < 20, RRT
Moriyama, 2016, Japan	Prospective observational	50/60	64 ± 13//65 ± 11	All	Elective on CPB	Emergent, EF 40%, > 80y, CKD
DeLoor, 2017, Belgium	Prospective observational	203/34.5	70 (61,76)	CABG, valve, combined, ao root	Elective	AKI, CKD 5, recent RTx, weekwnd surgery
Lee, 2019, Taiwan	Prospective observational	186/38	60 ± 14.6	All	Elective	CKD, organ Tx, RRT
Jie Hu, 2021, China	Retrospective observational of RCT	110/62	47.5 (43.25, 53.75)	Valve	Elective multiple valve on CPB	RRT
Sheng-Wen Ko, 2018, Taiwan	Prospective observational	151/33	63 ± 14	CABG, valve, aorta	CABG valve aorta	AKI, RRT, nephrectomy, RTx, GFR < 30
Liebetrau, 2013, Germany	Prospective observational	141/31	68 ± 11//74 ± 8	CABG, valve, combined	Elective CABG and valve on CPB	GFR < 30
McIlroy, 2015, USA	Prospective observational	603/37	65 ± 16//67 ± 14	All	All cardiac surgery, first opus Moday-Thursday	RRT
Fanning, 2016, New Zealand	Prospective observational	50/38	71 (38,87 Range)	CABG, valve, combined	High risk for CS-AKI, on CPB	GFR < 50, IABP, emergent, offpump, immunosuppression, bacteraemia, on steroids > 10 mg prednisolone
Metzger, 2016, Germany	Retrospective observational	110	60(24-77Range))//65(45-77Range	elective on CPB	elective on CPB	Emergent, hemodynamic instability
Liu, 2012, China	Prospective observational	109/34	63mean ± 11.3	CABG, valve, combined	Elective on and offpump	ESRF, RRT, emergent, death within 24 h
Matsui, 2012, Japan	Prospective observational	85/25	70median(17–86)//73 (36-87range)	CABG, valve, combined, aneurysm	Elective on and offpump	emergent, RRT. death in 24 h
Sargentini, 2012, Italy	Prospective observational	52/29	66.7 ± 11//74 ± 6	CABG, valve, combined	Elective on CPB	nd
Varela, 2015, Brazil	Prospective observational	66/26	68 ± 11	All	Cardiac surgery	AKI, ESRD, RTx, ionidated-contrast within 72 h
Wagener, 2006, USA	Prospective observational	81/34.6	67(49,76)//73(66–76)	CABG, valve, multiple valve, combined	CABG, valve, multiple valve, combined	CKD req RRT
Prowle, 2015, Australia	RCT	93/31	70 (61, 76)	CABG, valve, thoracic aorta (11%)	Elective on CPB	nd
JianJhongWang, 2018, Taiwan	Prospective observational	149/30	62 ± 14	CABG, valva, aortic	Cardiac surgery	AKI, RRT, nephrectomy, RTx, GFR < 30
Koyner, 2010, USA	Prospective observational	123/35	68 (57,77)//72(60, 81)	CABG, valve, combined, other	Elective	RRT, RTx, emergent, unstable renal function, oliguria
Parikh, 2011, USA	Prospective observational	1219/32	71 ± 10	VABG, valve, combined	High risk: 1of emergent, kidney impairment, EF < 35%, combined, redo, DM, > 70y)	preop AKI, RTx, SeCreat > 400, nephrotoxin, only VAD
Schley, 2015, Germany	Prospective observational	110/24	70 ± 10	CABG, valve, aorta, combined	onCPB	RRT, pregnancy, RTx
Miaolin Che, 2010, China	Retrospective observational	29/31	63 ± 14	CABG, valve, aorta, combined, CHD	elective	CKD
Liangos, 2009, USA	Prospective observational	103/28	68 ± 11	All	Onpump	Offpump, pregnancy, RRT, organ Tx in 1y
Paarman, 2013, Germany	Retrospective analysis of prospective data	136/27	-/63.7/71.8	CABG, valve, combined, other	Onpump	ESRD, offpump
Han, 2009, USA	Prospective observational	90	-/60/68	All	All cardiac surgery	RRT, death in 24 h
Albert, 2020, Germany	Ancilliary study of RCT	100/33	-/67(56–73)/74(70–74)	CABG, valve, combined	Elective onCPB	Emergent, offpump, SeCrea > 300, RTx, immunosuppresion
Heise, 2011, Germany	Prospective observational	47/26	69.5 ± 11	CABG, valve, combined, myxoma	Elective onCPB	RRT
Tidbury, 2019, UK	Post hoc analysis of RCT	125/46	-/75/74	CABG, valve, combined	High-risk, elective, on pump, eGFR < 60	CPB < 60 min, aortic surgery, liver impairment, eGFR < 15, RRT, malignancy, pregnancy
Elmedany, 2017, Egypt	Prospective observational	45/18		CABG	On pump, elective, CABG, CCS < 5	Redo, renal impairment, periop nephrotoxic drugs, > 75y, EF < 35%, emergency, combined procedure, neoplasm
Averdunk, 2019, Germany	Prospective observational	148/26		CABG, valve, combined	On pump, elective	Emergency, pregnancy, RRT
Sun, 2021, China	Prospective observational	52/38		CABG, aorta, valve, other	Onpump	AKI, chr kidney disease, RRT, malignancy, organ Tx, > 80y
Haase, 2014, Germany	Nested cohort of RCT	100		CABG, aorta, valve, other	High risk for CSAKI, on CPB	
Garcia-Alvarez, 2015, Spain	Prospective observational	288/35		All	All cs, off and on pump	RRT, RTx, coronarography in7days
Quian, 2019, China	Prospective observational	91/42	62 + −9	All	All cs, off and on pump	CKD, thyroid disease, high dose steroids, preop UTI
Xin, 2008, China	Prospective observational	33/42		All	All cs, off and on pump	Renal insuff, DM, ACEI, neprotoxins, unstable vital signs
Kar, 2021, Bangladesh	Prospective observational	42		CABG	CABG onpump	Renal dysfunction, NSAID, EF < 30
Wagener, 2008, USA	Prospective observational	426/34	63 + −15	CABG,valve,combined Htx, VAD, reop	All cardiac surgery	RRT
Wan, 2008, China	Prospective observational	33/42	-/40 + −20/37 + −20	CABG, valve	CABG, valve on CPB	CABG, valve on CPB
Munir, 2013, Pakistan	Prospective observational	88/14	52	Cardiovascular	On CPB	chr kidney disease,rtx, nephrotoxin
Mori, 2014, Japan	Prospective observational	36/25	71 (48–85)range	Ao arch with DHCA	Ao arch with DHCA	SeCreat > 106
TaoHanLee, 2021, Taiwan	Prospective observational	144/34	62 + −13	CABG, valve, aorta, combined	All cardiac surgery	< 20y,GFR < 30, RRT, organ transplant,preop AKI
Parikh, 2013, USA	Prospective observational	1219/32	71 + −10	CABG, valve, combined	High risk: 1of these:emergent, kidney impairment, EF < 35%, combined, redo, DM, > 70y	preop AKI, RTx, SeCreat > 400, nephrotoxin, only VAD
Katagiri, 2012, Japan	Prospective observational	77/39		OPCAB,valve, combined, other	Elective cardiac	ESRD, RtX
McIlroy, 2018, USA	Prospective observational	603/37		All	All cardiac, 1st op from Mon-Thur	RRT
Khreba, 2019, Egypt	Prospective observational	45/49	46 + −14.6	On CPB	On CPB, GFR > 90, normal kidney US	
Silverton, 2021, USA	Prospective observational	90/31		CABG, valve, combined, aorta, VAD	On CPB, high risk by CS	RRT, emergent sy, preop ECMO
Koyner, 2013, USA	Prospective observational	1203/32	71 + −10	CABG, valve, combined	High risk: 1of these:emergent, kidney impairment, EF < 35%, combined, redo, DM, > 70y	preop AKI, RTx, SeCreat > 400, nephrotoxin, only VAD
Susantitaphong, 2013, USA	Prospective observational	252/27	68	On pump	On pump	Offpump,, pregnancy, RRT, organTx
Shu, 2016, Taiwan	Prospective observational	141/30.5	62.4 + −13.6	CABG, valve	Cariovasc sy, CABG and valve	RRT, prev AKI,Rtx, nephrectomy, eGFR < 30
Yavuz, 2009, Turkey	Prospective observational	51/15.7		CABG	preop SeCreat 132.6–176.8, randomised to off and onpump	Oliguric/anuric chr kidney disease, EF < 30, recent surgery, nephrotoxins
Neyra, 2019, USA	Prospective observational	106/27.4	61 ± 12	CABG, valve	CABG, valve on CPB	< 20y, HIV pos, HCV pos, Htc < 25
Levante, 2017, Italy	Retrospective observational	110/31.9	69 (59–76)	All	All cardiac surgery	TAVI, hemodialysis
Ho, 2011, Canada	Prospective observational	338/22	63 + −10	cABG, valve,	Elective on CPB	AKI in 24 h
ChanganWang, 2017, China	Prospective observational	103/47.6	58/59	On CPB	No renal and hepatic dysfunction	Tumor, AKI, nephrotoxins, unstable vitals
Merchant, 2018, USA	Prospective observational	47/44.6		CABG, valve, both	Elective, high risk for AKI, Thakar > 5,	CKD 5, ESRD, oliguriapreop, pregnancy, unstable SeCret
Prowle, 2012, Australia	RCT	93/31	70 (61,76)	CABG, valve, thoracic aorta (11%)	elective on CPB	nd
Kambhampati,2013, USA	Prospective observational	100/40	61.4 + −1.4	TAA,valve, CABG	cardiovasc surgery, eGFR > 30	OrganTX, IABP, on natriuretic peptid
Haase, 2008, Australia	Prospective observational	100/39	68/75	CABG, valve, combined	On CPB	Emergency, offpump, ESRF, RTx
Kanchi, 2023, India	Prospective observational	90/10	53.49 ± 9.05	Elective OPCAB	Elective OPCAB	Onpump, emergent, CKD, on RRT, renal Tx
Monaco, 2024, multinational	2nd analysis of RCT	337/37.7	63 [52–72]	All cardiac	Elective cardiac surgery	AKI, CKD, pregnancy, RRT, ECMO, IABP
Lacquaniti, 2023, Italy	Prospective observational	230/37	65.3 ± 7.9	CABG	Adult CABG	Kidney insufficiency, ECMO, VAD, IABP
Yun, 2024, Italy	Prospective observational	557/29.4	66.4 ± 11.8	Cardiac surgery	Adult cardiac surgery	preop AKI, CKD stage 4–5
Ghaheh, 2021, Iran	Cross sectional	96/29.2	48.88 ± 4.52/ 50.04 ± 6.02	CABG	On CPB	BMI > 40, CKD, liver failure, drugs,coag problem, offpump
Chica, 2024, Switzerland	Prospective observational	44/59	65.6 ± 2.31/71.7 ± 1.64	On CPB	On CPB	Renal dysfunction
Fang, 2022, China	Prospective observational	621/19.3	52.8 ± 11.6	TAAR	TAAR	ESRD, RtX, nephrectomy, pregnancy, RRT
Takaki, 2024, Japan	Prospective observational	111/22.5	70 [61–74]	OPCAB	OPCAB	On-pump
Udzik, 2022, Poland	Prospective observational	128/29.7	67 (67–75)/70 (61–72)	All cardiac	elective	ESRD,CKD, active inflammatory disease, active neoplasm

We collected our data mainly from articles on prospective observational trials. We summarized additional data on urinary biomarker measurements in the included articles in Supplementary Table 1.

First, we investigated individual urinary biomarkers regarding predictive accuracy; second, we pooled AUC-ROC values of different combinations of urinary biomarkers for predicting CS-AKI. Third, we could analyse clinical scores and clinical model performance without and with the addition of urinary biomarker measurements.

### Primary outcome

#### Individual biomarkers

None of the individual biomarkers achieved excellent prediction values.

To predict all stages of CS-AKI as defined by the KDIGO criteria, reports on TIMP2xIGFBP7, NGAL, and KIM-1 could be analysed; the pooled AUC-ROC values measured at different time points are shown in Table 2.

**Table 2:** Individual biomarker prediction of all AKI and severe AKI defined by KDIGO criteria at different measurement time-points

	Preoperative	Intraoperative and early postoperative	From postoperative 6 h to postoperative day 1
All AKI (a)			
TIMP2xIGFBP7	0.55 [0.47, 0.62]	0.66 [0.62, 0.71]	0.64 [0.62, 0.66]
NGAL	nd	0.69 [0.65, 0.73]	0.66 [0.64, 0.68]
KIM-1	nd	0.62 [0.58, 0.67]	nd
Severe AKI (b)			
TIMP2xIGFBP	0.59 [0.53,0.65]	0.75 [0.69, 0.81]	0.74 [0.66, 0.83]
NGAL	nd	0.73 [0.65, 0.82]	0.66 [0.64, 0.68]

Pooled AUCs with 95% confidence intervals; subgroup analyses based on merged measurement time-points; *TIMP2xIGFBP7* tissue inhibitor metalloproteinase 2 and insulin like growth factor binding protein 7, *KIM-1* kidney injury molecule 1, *NGAL* neutrophil gelatinase associated lipocalin

### Secondary outcomes

#### Individual biomarkers

The pooled AUC-ROC values for articles investigating TIMP2xIGFBP7 and NGAL prediction of severe AKI defined by KDIGO criteria are shown in Table 2.

To predict all stages of AKI defined by any diagnostic criteria (KDIGO, AKIN, and RIFLE), the pooled AUC values of NGAL, L-FABP, IL-18, KIM-1, α-GST, normalised Cystatin-C, and normalised NAG are shown in Table 3, pooled AUCs for the identification of severe AKI cases are shown in Table 3.

**Table 3:** Individual biomarker prediction for all AKI and severe AKI defined by all criteria at different time-points

	Preoperative	Intraoperative and early postoperative	From postoperative 6 h to postoperative day 1	Postoperative day 2–3
ALL AKI (a)				
α-GST	nd	0.57 [0.55, 0.60]	nd	nd
Norm CysC	nd	0.63 [0.54, 0.73]	nd	nd
IL-18	nd	0.73 [0.54, 0.92]	0.56 [0.48, 0.64]	nd
KIM-1	0.64 [0.50, 0.78]	0.63 [0.59, 0.67]	0.66[0.54, 0.79]	nd
L-FABP	nd	0.75 [0.67, 0.82]	0.71 [0.64, 0.79]	nd
NGAL	0.60 [0.54,0.67]	0.71 [0.67, 0.76]	0.68 [0.64, 0.71]	0.65 [0.54, 0.76]
Norm NAG	nd	0.67 [0.56, 0.77]	nd	nd
Severe AKI (b)				
α-GST	nd	0.52 [0.47, 0.57]	0.54 [0.42, 0.66]	nd
L-FABP	nd	0.70 [0.56, 0.84]	nd	nd
NGAL	nd	0.74 [0.67, 0.80]	0.71 [0.61, 0.81]	nd
π-GST	nd	0.74 [0.72, 0.77]	0.63 [0.58, 0.67]	nd

Legend: Pooled AUCs with 95% confidence intervals; subgroup analyses based on merged measurement time-points; *α- and π-GST* α-and π-glutathione-S-transferase, *KIM-1* kidney injury molecule 1, *NAG* N-acetyl-β-D-glucosaminidase, *IL-18* interleukin-18, *NGAL* neutrophil gelatinase-associated lipocalin, *CysC* cystatin C, *L-FABP* L-type fatty acid binding protein

Normalising urinary biomarkers with urinary creatinine concentration significantly improved the predictive value of KIM-1 measured on postop days 2 and 3 (p = 0.0321). However, this did not affect the performance of any other biomarker. For Cystatin-C and NAG, we could only pool data on normalised biomarkers; these are accordingly shown in Table 3. For π-GST, normalization reduced accuracy (p = 0.0862).

Our pooled data suggest that each biomarker gives the best predictive value when measured in the intraoperative or early postoperative period; however, this increase in accuracy is not significant.

**Fig. 2:**
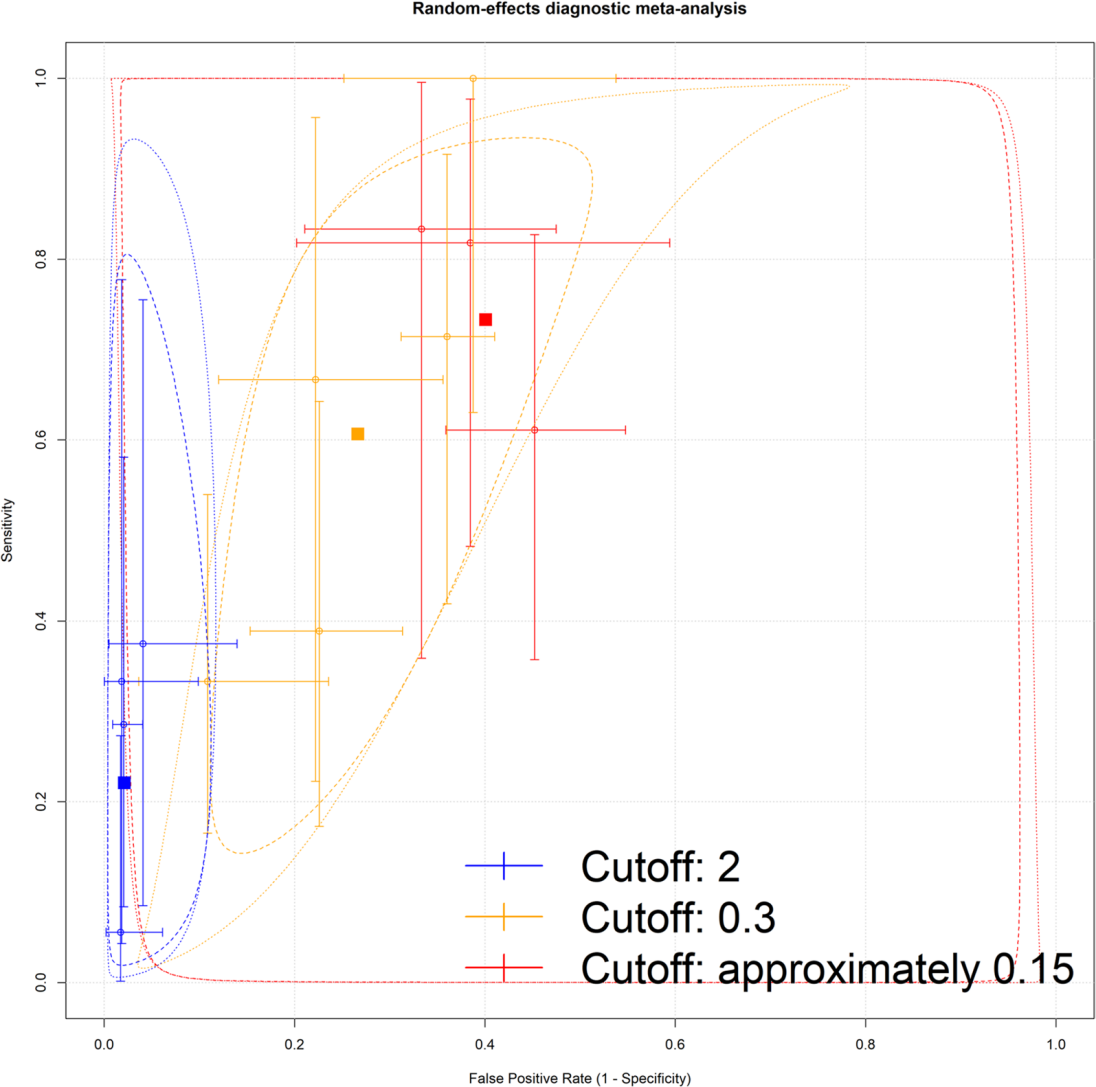
Pooled sensitivity and specificity of TIMP2xIGFBP7 for CS-AKI prediction by different thresholds

We had sufficient data to calculate the pooled sensitivity and specificity for TIMP2xIGFBP7 at the two widely used thresholds: threshold 2.0 (ng/ml)^2^/1000 provided excellent specificity of 98.76% but poor sensitivity of 40%, whereas threshold 0.3 (ng/ml)^2^/1000 had a sensitivity of 81% and specificity of 82%. Our results suggest that the threshold of 0.15 (ng/ml)^2^/1000 yielded a sensitivity of 87% and a specificity of 68%, as presented on Fig. 2.

#### Combination of urinary biomarkers

Sixteen authors also reported on the performance of combinations of individual biomarkers; however, calculations of combined biomarker AUCs showed significant heterogeneity, and most of the information on mathematical methods was lacking (Supplementary ref 1,2,7,11,12,13,14,15,16,17,18,19,20,21,22,23).

**Fig. 3:**
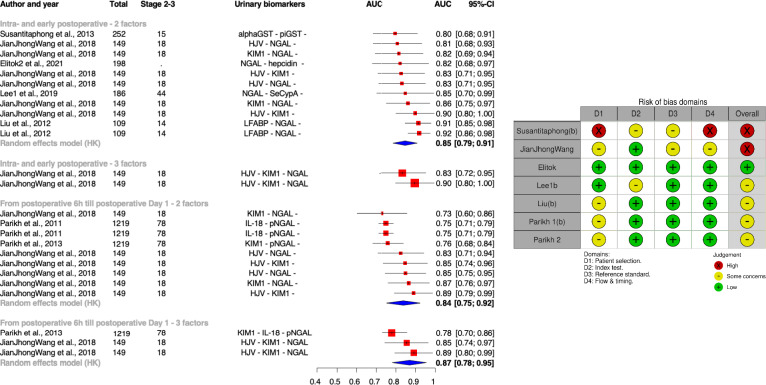
Combination of biomarkers for prediction of stages 2 and 3 acute kidney injury. Risk of bias assessment shown on the right; *CS-AKI* cardiac surgery related acute kidney injury, *Alpha and piGST* alpha and pi gluthation-S-transferase, *KIM-1* kidney injury molecule 1, *NAG* N-acetyl-β-D-glucosaminidase, *IL-18* interleukin-18, *NGAL* neutrophil gelatinase-associated lipocalin, *CysC* cystatin C, *CypA* cyclophilin A, *L-FABP* L-type fatty acid binding protein, *RBP* RNA binding protein, *HJV* hemojuvelin, *pNGAL* plasma neutrophil gelatinase-associated lipocalin

We pooled these data regardless of the applied mathematical methods as shown in Fig. 3.

As for the combination of biomarkers, the pooled AUC-ROC for all stages and severe AKI was 0.82 [0.75, 0.88] and 0.85 [0.79, 0.91], respectively, for intra- and early postoperative measurements. The number of combined biomarkers did not improve accuracy; AUC was 0.84 for two combined UBs and 0.87 for more than two combined UBs (p = 0.6247).

#### Predictive value of clinical models combined with urinary biomarkers

**Fig. 4:**
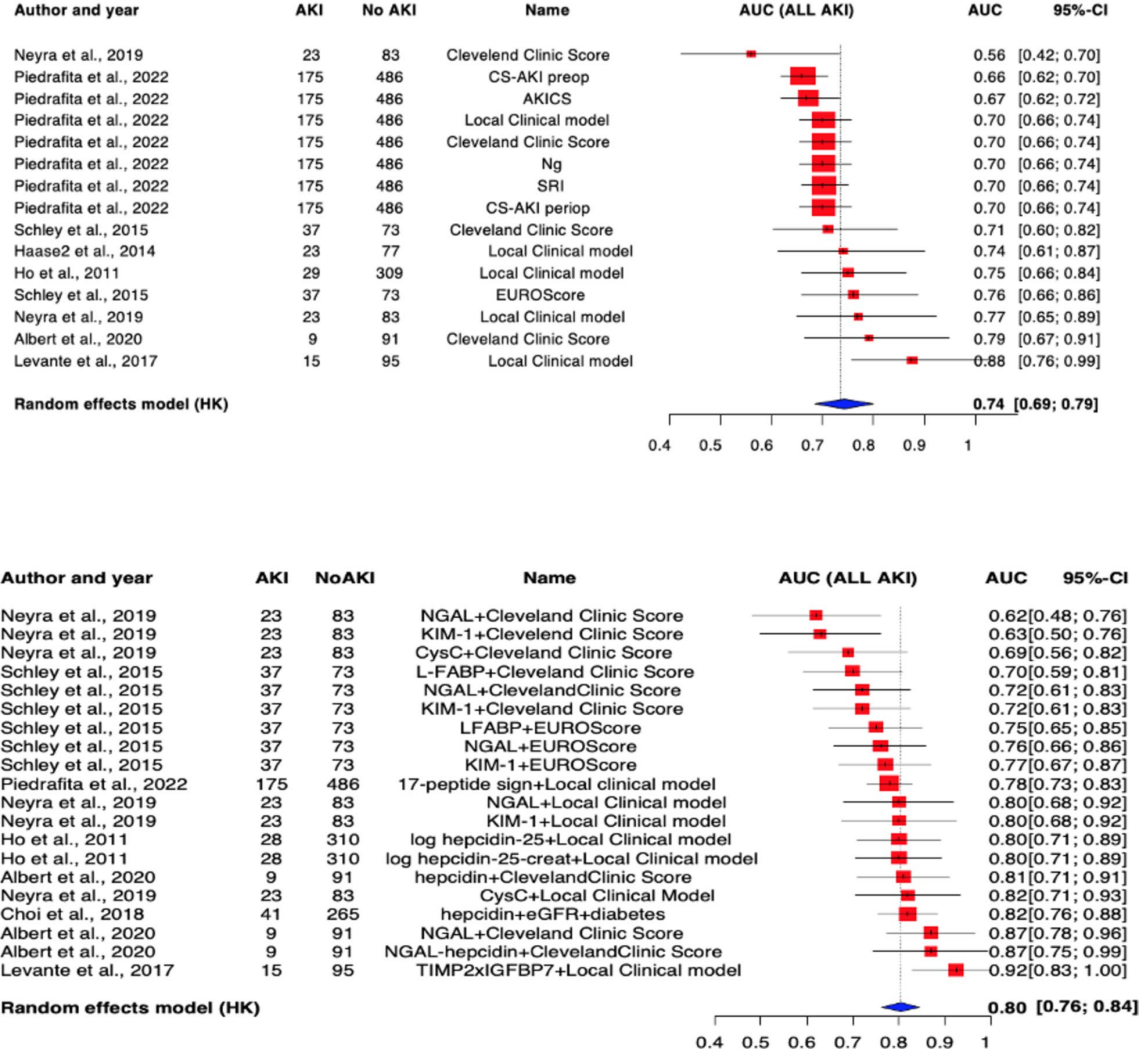
Prediction of all stages of acute kidney injury by clinical models and urinary biomarkers. Top: clinical scoring systems alone. Bottom: Scoring systems enhanced by urinary biomarkers. Better predictive value, but non-significant difference p = 0.0666. Acute kidney injury prediction scores: Cleveland Clinic Score, CS-AKI preop and periop, AKICS, Ng, SRIMortality score: EUROScore; Urinary biomarkers: *NGAL* neutrophil gelatinase associated lipocalin, *KIM-1* kidney injury molecule-1, *CysC* cystatin C, *L-FABP* L-type fatty acid binding protein, *TIMP2xIGFBP7* tissue inhibitor metalloprotease-2 insulin growth factor binding protein

Altogether nine articles reported on the accuracy of clinical scores (Cleveland Clinic Score, EuroScore) and locally developed clinical prediction models and the increase of predictive values when a urinary biomarker measurement was added (Supplementary ref 22, 24, 25, 26, 27, 28, 29, 30, 31). The pooled AUC of clinical prediction models and scores was 0.74 [0.69, 0.79] and for models improved by urinary biomarker measurements was 0.80 [0.76, 0.84], as shown in Fig. 4. However, the improvement was not significant.

### Risk of bias and GRADE assessment

The results of the risk of bias assessment are presented in Supplementary Table 2. Overall, the risk of bias was moderate to high, as 40% of the studies used frozen urine samples analysed in batches and the time of measurement of biomarker levels was not reported, nor was the application of blinding to clinical outcomes of laboratory and research personnel.

The certainty of evidence proved to be low for all outcomes due to the study design. GradePro assessment can be found in the Supplementary Table 3.

### Qualitative synthesis: other biomarkers

Urinary biomarkers with less than three reports include hemojuvelin, angiotensinogen, neprilysin, microRNA, ATF3 and trace elements. Two authors investigated Hemojuvelin in overlapping populations, with AUCs ranging from 0.71 to 0.91 to predict severe AKI (Supplement ref 32, 33). Angiotensinogen was also examined by two authors, yielding poor predictive value (Supplement ref 34, 35). Neprilysin, a glycoprotein located at the renal brush border, may have the potential as an AKI biomarker based on a recent article reporting an AUC of 0.77 (Supplement ref 36). Two studies reported on the performance of microRNA, one for microRNA-21 with an AUC of 0.9 at 6 h post-surgery and one for miR-125a-5p with an AUC of less than 0.60 (Supplement ref 37,38). MicroRNAs are short oligonucleotides that regulate post-transcriptional gene expression, and several contribute to AKI. ATF3 (urinary exosomal activating transcriptional factor 3) is a key regulator of the stress response and has been shown to play a role in ischemia–reperfusion injury and sepsis-induced AKI. Wu et al. reported an AUC of 0.691 to diagnose AKI 12 h postoperatively (Supplement ref 39). Trace elements have recently been investigated by Gardner et al., who found a difference between AKI predictor trace elements in cardiac surgery and general ICU patient populations (Supplement ref 40). Zn had a value of an AUC of 0.77 in cardiac surgical patients after surgery. In contrast, in the general ICU population, Zn, Cd and Cu also had fair predictive values. These molecules and elements offer a possible future research path for detecting more accurate urinary biomarkers.

### Qualitiative synthesis: other outcomes

Deviating from our registered study protocol, we opted out of meta-analysing length of stay and mortality due to the high between-study heterogeneity and poor evidence quality.

Forty-three articles report on length of stay and mortality outcomes. Both ICU and hospital length of stay are much longer in the CS-AKI patient groups, a well-known phenomenon in the critically ill patient population.

Reporting mortality outcomes is not homogenous, with follow-up times ranging from 30 days to 1 year, including composite outcomes (mortality or RRT, major adverse kidney events (MAKE) and all-cause mortality. Most studies show elevated death rates in the AKI groups. Brown et al. investigated 30-day and 365-day readmission and death rates. They concluded that urinary biomarkers were not associated with readmission and mortality, whereas AKI duration of more than 3 days was strongly predictive (Supplementary ref 41).

## Discussion

In the current systematic review and meta-analysis, we reviewed 95 articles, including 14,455 patients who underwent CS. Our main findings are that individual urinary biomarkers may identify patients developing CS-AKI with acceptable accuracy, but combining urinary biomarkers or clinical models combined with urinary biomarkers can result in excellent accuracy.

### Prediction of CS-AKI by individual urinary biomarkers

Our results show that the prediction of CS-AKI by measuring individual urinary biomarkers gives acceptable accuracy, with the highest performance when measured in the intraoperative or early postoperative period. For prediction of all stages of CS-AKI, IL-18 and L-FABP give the highest accuracy, and for early identification of patients developing severe CS-AKI, NGAL, TIMP2xIGFBP7 and π-GST are the most accurate. TIMP2xIGFBP7 can be measured with a bedside test (Nephrocheck), which was applied by Zarbock et al. in the PrevAKI trial to identify patients at high risk of KDIGO care-bundle intervention to reduce the incidence of CS-AKI [5]. Measurements of other biomarkers mentioned above are performed in laboratories, take longer, and are less useful in everyday practice. Cummings et al. performed serial measurements of TIMP2xIGFBP7 and obtained an excellent predictive value for CS-AKI, and demonstrated that elevation of serum creatinine occurs later than the increase of urinary biomarkers [25]. An increase in renal stress urinary biomarkers already signals early renal stress when the loss of function and the consequential elevation of creatinine is not apparent [25]. A recent meta-analysis of eight articles by Tai et. al found an AUC of 0.868 for the prediction of CS-AKI by TIMP2xIGFBP7 [26]. However, our review of 19 publications on TIMP2xIGFBP7 found that intraoperative and early postoperative measurements yielded only acceptable accuracy [24], (Supplement ref 24, 30, 42–57).

Currently, two thresholds are used for TIMP2xIGFBP7 measurements, 0.3 (ng/ml)^2^/1000 and 2.0 (ng/ml)^2^/1000. Our analysis suggests that a lower threshold of 0.15 (ng/ml)^2^/1000 may be applied to achieve maximum sensitivity in screening for severe cases of CS-AKI, even at the cost of reducing specificity, given the significant potential harm of preventive measures.

### Prediction of CS-AKI by combination of urinary biomarkers

Our study is the first to analyse the combination of urinary biomarkers in predicting CS-AKI. We pooled all potential UB combinations available in reports, regardless of biomarker types and calculation methods. We aimed to investigate the predictive value of any combination of individual UBs already examined. The combination of urinary biomarkers resulted in good accuracy. Increasing the number of molecules measured does not lead to a better predictive value, but it may increase healthcare costs. Therefore, we do not recommend using more than two UB measurements to predict CS-AKI.

### Prediction of CS-AKI by clinical models enhanced with urinary biomarkers

The prediction of CS-AKI based on clinical models and scoring systems is widespread in bedside practice worldwide. Several scoring systems have been established over the past two decades (EuroScore, Cleveland Clinic Score, and AKICS); however, their predictive value does not achieve the excellent range [27–29]. Locally developed clinical multi-regression models have also been published with varying accuracy (Supplement ref 25, 26). In our analysis of nine reports, we focused on the predictive value of any scoring system and the potential effect of urinary biomarker incorporation on accuracy. We found that the predictive value of an established or locally developed clinical model increases from acceptable to good accuracy with the addition of a urinary biomarker measurement, but not to a statistically significant degree. By continuing to use well-known scoring systems and available urinary biomarker measurements on-site, clinicians can achieve an excellent identification rate of high-risk patients for CS-AKI.

### Strengths and limitations

To the best of our knowledge, this is one of the largest and most comprehensive meta-analyses investigating the predictive value of urinary biomarkers in the field of CS-AKI to date. It includes predictive values for individual urinary biomarkers in CS-AKI and the combination of biomarkers and prediction models. We included articles using all acute kidney injury criteria (KDIGO, RIFLE, and AKIN) and performed subgroup analysis based on criteria that showed no difference in pooled AUCs. The RIFLE criteria generally over-diagnose AKI by the RIFLE-Risk category [30]. Articles using RIFLE were included in the study of severe AKI outcomes, where RIFLE-Injury and RIFLE-Failure categories corresponded to those of AKIN and KDIGO Stages 2 and 3. We, therefore, decided to analyse articles with different AKI criteria together.

Our review has certain limitations, mostly due to the heterogeneity of the articles included. Urinary biomarker measurement time points show a high variability across studies, forcing us to merge time points based on pathophysiologic considerations and perform subgroup analysis accordingly. The high level of heterogeneity arising from both clinical parameters and methodological sources limits the generalizability of our findings on the patient level. Evidence supporting the utilization of the investigated biomarkers are equally affected and diminished by this limitation. We observed a high risk of bias among studies due to the non-universal reporting techniques. The earliest published articles included date back to 2008, and there have been changes in reporting regulations, which may increase the risk of bias in earlier studies. In addition, studies before 2015 mainly reported on urinary biomarkers measured in batches after the study observation period, which inherently introduced bias. More recent publications benefit from the advantages of bedside tests or faster laboratory techniques. The article population is homogeneous regarding extrinsic factors of AKI, i.e., patients undergoing cardiac surgery (more than 70% on CPB); however, intrinsic factors show high heterogeneity, as patients are included irrespective of preoperative renal function and predisposing co-morbidities.

Publication bias could not be assessed using conventional methods, such as funnel plots due to the complexity of the multivariate mixed effects model used.

### Implications for practice and research

We recommend using urinary biomarkers in combination or as an addition to clinical prediction scores to identify patients developing for CS-AKI at an early stage, when preventive measures can be implemented. We recommend using NGAL, TIMP2xIGFBP7, and pi-GST for individual urinary biomarker measurements to identify patients developing severe CS-AKI. Our results suggest that researchers should report on urinary biomarkers to describe the exact times of measurements and blind personnel to clinical data to reduce the risk of bias. Given the extensive data, we do not recommend further research into the accuracy of already established urinary biomarkers. However, we recommend research into new, stable and easy-to-measure biomarkers.

The current study was conducted to accelerate the implementation of evidence-based medicine in healthcare practices, and to bridge the gap between clinical practice and medical science, in line with the goals of translational medicine [31, 32].

## Conclusion

Individual urinary biomarkers can identify patients developing CS-AKI with acceptable accuracy. Combining urinary biomarkers and clinical models improved with urinary biomarkers provides good accuracy for predicting CS-AKI, allowing physicians to diagnose individuals at risk early and implement preventive measures to reduce the incidence and progression of CS-related AKI.

## Supplementary Information

Supplementary material 1.

Supplementary material 2.

## Data Availability

The datasets used in this study can be found in the full-text articles included in the systematic review and meta-analysis.

## Abbreviations

ADQI: Acute disease quality initiative
AKI: Acute kidney injury
AKIN: Acute kidney injury network
ATF3: Urinary exosomal activating transcriptional factor 3
α-GST: α-Glutathione-S-transferase
AUC: Area under the receiving operator curve
AUC-ROC: Area under the receiving operator curve
CI: Confidence interval
CPB: Cardio-pulmonary bypass
CS: Cardiac surgery
CS-AKI: Cardiac surgery-associated kidney injury
ERAS: Enhanced recovery after surgery
FN: False negative
FP: False positive
GFR: Glomerular filtration rate
ICU: Intensive care unit
IL-18: Interleukin-18
KDIGO: Kidney Disease Improving Global Outcomes
KIM-1: Kidney injury molecule-1
L-FABP: Liver-type fatty acid binding protein
LR: Likelihood ratio
MAKE: Major adverse kidney events
MicroRNA: Micro-ribonucleic acid
NAG: N-Acetyl-β-D-glucosaminidase
NGAL: Neutrophil gelatinase-associated lipocalin
NPV: Negative predictive value
π-GST: π-Glutathione-S-transferase
PPV: Positive predictive value
PRISMA: Reporting items for systematic reviews and meta-analyses
RIFLE: Risk, injury, failure, loss, end-stage renal disease
RRT: Renal replacement therapy
SE: Standard error
sCR: Serum creatinine
TIMP2xIGFBP7: Tissue-inhibitor of metalloproteinase-2 and insulin growth factor binding protein-7
TN: True negative
TP: True positive
UB: Urinary biomarker
UBs: Urinary biomarkers

## References

[1] Lei C, Berra L, Rezoagli E, et al. Nitric oxide decreases acute kidney injury and stage 3 chronic kidney disease after cardiac surgery. Am J Respir Crit Care Med. 2018;198(10):1279–87. 29932345 10.1164/rccm.201710-2150OC PMC6290943

[2] Hobson CE, Yavas S, Segal MS, et al. Acute kidney injury is associated with increased long-term mortality after cardiothoracic surgery. Circulation. 2009;119(18):2444–53. 19398670 10.1161/CIRCULATIONAHA.108.800011

[3] Kellum JA, Lameire N; KDIGO AKI Guideline Work Group. Diagnosis, evaluation, and management of acute kidney injury: a KDIGO summary (Part 1). Crit Care. 2013;17(1):204. 10.1186/cc11454 PMC4057151 23394211

[4] Ronco C, Rosner MH. Acute kidney injury and residual renal function. Crit Care. 2012;16(4):144. 22866976 10.1186/cc11426 PMC3580707

[5] Zarbock A, Küllmar M, Ostermann M, et al. Prevention of cardiac surgery-associated acute kidney injury by implementing the KDIGO guidelines in high-risk patients identified by biomarkers: the PrevAKI-Multicenter randomized controlled trial. Anesth Analg. 2021;133(2):292–302. 33684086 10.1213/ANE.0000000000005458

[6] Peng K, McIlroy D, Bollen BA, et al. Society of cardiovascular anesthesiologists clinical practice update for management of acute kidney injury associated with cardiac surgery. Anesth Analg. 2022;135(4):744–56. 35544772 10.1213/ANE.0000000000006068

[7] Ostermann M, Zarbock A, Goldstein S, et al. Recommendations on acute kidney injury biomarkers from the acute disease quality initiative consensus conference: a consensus statement. JAMA Netw Open. 2020;3(10):e2019209. 33021646 10.1001/jamanetworkopen.2020.19209

[8] Engelman DT, Ben Ali W, Williams JB, et al. Guidelines for perioperative care in cardiac surgery: enhanced recovery after surgery society recommendations. JAMA Surg. 2019;154(8):755–66. 31054241 10.1001/jamasurg.2019.1153

[9] Page MJ, McKenzie JE, Bossuyt PM, et al. The PRISMA 2020 statement: an updated guideline for reporting systematic reviews. BMJ. 2021;372:n71. 33782057 10.1136/bmj.n71 PMC8005924

[10] Cochrane Handbook for Systematic Reviews of Interventions Version 6.4, 2023

[11] Whiting PF, Rutjes AW, Westwood ME, et al. QUADAS- A revised tool for the quality assessment of diagnostic accuracy studies. Ann Intern Med. 2011;155(8):529–36. 22007046 10.7326/0003-4819-155-8-201110180-00009

[12] GRADE pro [cited 18 December 2024] https://www.gradepro.org/.

[13] Freeman SC, Kerby CR, Patel A, Cooper NJ, Quinn T, Sutton AJ. Development of an interactive web-based tool to conduct and interrogate meta-analysis of diagnostic test accuracy studies: MetaDTA. BMC Med Res Methodol. 2019;19(1):81. 30999861 10.1186/s12874-019-0724-x PMC6471890

[14] Hanley JA, McNeil BJ. The meaning and use of the area under a receiver operating characteristic (ROC) curve. Radiology. 1982;143(1):29–36. 7063747 10.1148/radiology.143.1.7063747

[15] Pustejovsky JE, Tipton E. Meta-analysis with Robust variance estimation: expanding the range of working models. Prev Sci. 2022;23(3):425–38. 33961175 10.1007/s11121-021-01246-3

[16] Rutter CM, Gatsonis CA. A hierarchical regression approach to meta-analysis of diagnostic test accuracy evaluations. Stat Med. 2001;20(19):2865–84. 11568945 10.1002/sim.942

[17] Reitsma JB, Glas AS, Rutjes AW, Scholten RJ, Bossuyt PM, Zwinderman AH. Bivariate analysis of sensitivity and specificity produces informative summary measures in diagnostic reviews. J Clin Epidemiol. 2005;58(10):982–90. 16168343 10.1016/j.jclinepi.2005.02.022

[18] Chu H, Cole SR. Bivariate meta-analysis of sensitivity and specificity with sparse data: a generalized linear mixed model approach. J Clin Epidemiol. 2006;9(12):1331–3. 10.1016/j.jclinepi.2006.06.011 17098577

[19] Harbord RM, Deeks JJ, Egger M, Whiting P, Sterne JA. A unification of models for meta-analysis of diagnostic accuracy studies. Biostatistics. 2007;8(2):239–51. 16698768 10.1093/biostatistics/kxl004

[20] Burke DL, Ensor J, Snell KIE, van der Windt D, Riley RD. Guidance for deriving and presenting percentage study weights in meta-analysis of test accuracy studies. Res Synth Methods. 2018;9(2):163–78. 29115060 10.1002/jrsm.1283

[21] Hajian-Tilaki K. Receiver operating characteristic (ROC) curve analysis for medical diagnostic test evaluation. Caspian J Intern Med. 2013;4(2):627–35. 24009950 PMC3755824

[22] White N, Parsons R, Collins G, et al. Evidence of questionable research practices in clinical prediction models. BMC Med. 2023;21:339. 37667344 10.1186/s12916-023-03048-6 PMC10478406

[23] Mehta RL, Kellum JA, Shah SV, et al. Acute kidney injury network: report of an initiative to improve outcomes in acute kidney injury. Crit Care. 2007;11(2):R31. 17331245 10.1186/cc5713 PMC2206446

[24] Bellomo R, Ronco C, Kellum JA, Mehta RL, Palevsky P, Acute Dialysis Quality Initiative workgroup. Acute renal failure - definition, outcome measures, animal models, fluid therapy and information technology needs: the Second International Consensus Conference of the Acute Dialysis Quality Initiative (ADQI) Group. Crit Care. 2004;8(4):R204–12. 15312219 10.1186/cc2872 PMC522841

[25] Cummings JJ, Shaw AD, Shi J, Lopez MG, O’Neal JB, Billings FT 4th. Intraoperative prediction of cardiac surgery-associated acute kidney injury using urinary biomarkers of cell cycle arrest. J Thorac Cardiovasc Surg. 2019;157(4):1545-1553.e5. 30389130 10.1016/j.jtcvs.2018.08.090 PMC6431272

[26] Tai Q, Yi H, Wei X, et al. The accuracy of urinary TIMP-2 and IGFBP7 for the diagnosis of cardiac surgery-associated acute kidney injury: a systematic review and meta-analysis. J Intensive Care Med. 2020;35(10):1013–25. 30376758 10.1177/0885066618807124

[27] Nashef SA, Roques F, Sharples LD, et al. EuroSCORE II. Eur J Cardiothorac Surg. 2012;41(4):734–45. 22378855 10.1093/ejcts/ezs043

[28] Thakar CV, Arrigain S, Worley S, Yared JP, Paganini EP. A clinical score to predict acute renal failure after cardiac surgery. J Am Soc Nephrol. 2005;16(1):162–8. 15563569 10.1681/ASN.2004040331

[29] Palomba H, de Castro I, Neto AL, Lage S, Yu L. Acute kidney injury prediction following elective cardiac surgery: AKICS Score. Kidney Int. 2007;72(5):624–31. 17622275 10.1038/sj.ki.5002419

[30] Yaqub S, Hashmi S, Kazmi MK, Aziz Ali A, Dawood T, Sharif H. A comparison of AKIN, KDIGO, and RIFLE definitions to diagnose acute kidney injury and predict the outcomes after cardiac surgery in a South Asian cohort. Cardiorenal Med. 2022;12(1):29–38. 35240595 10.1159/000523828

[31] Hegyi P, Petersen OH, Holgate S, et al. Academia Europaea position paper on translational medicine: the cycle model for translating scientific results into community benefits. J Clin Med. 2020;9(5):1532. 32438747 10.3390/jcm9051532 PMC7290380

[32] Hegyi P, Erőss B, Izbéki F, Párniczky A, Szentesi A. Accelerating the translational medicine cycle: the academia Europaea pilot. Nat Med. 2021;27(8):1317–9. 34312557 10.1038/s41591-021-01458-8

