# Supplementary material for: Combination of urinary biomarkers can predict cardiac surgery-associated acute kidney injury: a systematic review and meta-analysis
Source: Ann Intensive Care. 2025 Mar 29;15:45. doi: 10.1186/s13613-025-01459-7 (PMC11953499; doi:10.1186/s13613-025-01459-7)
Supplement: Supplementary file 1 — Supplementary material 1. [file 13613_2025_1459_MOESM1_ESM.docx]

**SUPPLEMENTARY REFERENCES**

1. Parikh CR, Coca SG, Thiessen-Philbrook H, et al. Postoperative biomarkers predict acute kidney injury and poor outcomes after adult cardiac surgery. J Am Soc Nephrol. 2011;22(9):1748-1757.
2. Parikh CR, Thiessen-Philbrook H, Garg AX, et al. Performance of kidney injury molecule-1 and liver fatty acid-binding protein and combined biomarkers of AKI after cardiac surgery. Clin J Am Soc Nephrol. 2013;8(7):1079-1088.
3. Haase M, Bellomo R, Albert C, et al. The identification of three novel biomarkers of major adverse kidney events. Biomark Med. 2014;8(10):1207-1217.
4. Haase M, Bellomo R, Story D, Davenport P, Haase-Fielitz A. Urinary interleukin-18 does not predict acute kidney injury after adult cardiac surgery: a prospective observational cohort study. Crit Care. 2008;12(4):R96.
5. Haase-Fielitz A, Mertens PR, Plass M, et al. Urine hepcidin has additive value in ruling out cardiopulmonary bypass-associated acute kidney injury: an observational cohort study. Crit Care. 2011;15(4):R186.
6. Haase-Fielitz A, Plaß M, Kuppe H, et al. Low preoperative hepcidin concentration as a risk factor for mortality after cardiac surgery: a pilot study. J Thorac Cardiovasc Surg. 2013;145(5):1380-1386.
7. Prowle JR, Calzavacca P, Licari E, et al. Combination of biomarkers for diagnosis of acute kidney injury after cardiopulmonary bypass. Ren Fail. 2015;37(3):408-416.
8. Prowle JR, Ostland V, Calzavacca P, et al. Greater increase in urinary hepcidin predicts protection from acute kidney injury after cardiopulmonary bypass. Nephrol Dial Transplant. 2012;27(2):595-602.
9. McIlroy DR, Farkas D, Pan K, Pickering JW, Lee HT. Combining Novel Renal Injury Markers with Delta Serum Creatinine Early after Cardiac Surgery and Risk-Stratification for Serious Adverse Outcomes: An Exploratory Analysis. J Cardiothorac Vasc Anesth. 2018;32(5):2190-2200.
10. McIlroy DR, Farkas D, Matto M, Lee HT. Neutrophil gelatinase-associated lipocalin combined with delta serum creatinine provides early risk stratification for adverse outcomes after cardiac surgery: a prospective observational study. Crit Care Med. 2015;43(5):1043-1052.
11. Wang JJ, Chi NH, Huang TM, et al. Urinary biomarkers predict advanced acute kidney injury after cardiovascular surgery. Crit Care. 2018;22(1):108.
12. Susantitaphong P, Perianayagam MC, Tighiouart H, Kouznetsov D, Liangos O, Jaber BL. Urinary α- and π-glutathione s-transferases for early detection of acute kidney injury following cardiopulmonary bypass. Biomarkers. 2013;18(4):331-337.
13. Hu J, Rezoagli E, Zadek F, Bittner EA, Lei C, Berra L. Free Hemoglobin Ratio as a Novel Biomarker of Acute Kidney Injury After On-Pump Cardiac Surgery: Secondary Analysis of a Randomized Controlled Trial. Anesth Analg. 2021;132(6):1548-1558.
14. De Loor J, Herck I, Francois K, et al. Diagnosis of cardiac surgery-associated acute kidney injury: differential roles of creatinine, chitinase 3-like protein 1 and neutrophil gelatinase-associated lipocalin: a prospective cohort study. Ann Intensive Care. 2017;7(1):24.
15. Lee CC, Chang CH, Cheng YL, et al. Diagnostic Performance of Cyclophilin A in Cardiac Surgery-Associated Acute Kidney Injury. J Clin Med. 2019;9(1):108.
16. Elitok S, Kuppe H, Devarajan P, et al. Urinary Neutrophil Gelatinase-Associated Lipocalin/Hepcidin-25 Ratio for Early Identification of Patients at Risk for Renal Replacement Therapy After Cardiac Surgery: A Substudy of the BICARBONATE Trial. Anesth Analg. 2021;133(6):1510-1519.
17. Che M, Xie B, Xue S, et al. Clinical usefulness of novel biomarkers for the detection of acute kidney injury following elective cardiac surgery. Nephron Clin Pract. 2010;115(1):c66-c72.
18. Katagiri D, Doi K, Honda K, et al. Combination of two urinary biomarkers predicts acute kidney injury after adult cardiac surgery. Ann Thorac Surg. 2012;93(2):577-583.
19. Liangos O, Tighiouart H, Perianayagam MC, et al. Comparative analysis of urinary biomarkers for early detection of acute kidney injury following cardiopulmonary bypass. Biomarkers. 2009;14(6):423-431.
20. Elmedany SM, Naga SS, Elsharkawy R, Mahrous RS, Elnaggar AI. Novel urinary biomarkers and the early detection of acute kidney injury after open cardiac surgeries. J Crit Care. 2017;40:171-177.
21. Han WK, Wagener G, Zhu Y, Wang S, Lee HT. Urinary biomarkers in the early detection of acute kidney injury after cardiac surgery. Clin J Am Soc Nephrol. 2009;4(5):873-882.
22. Albert C, Haase M, Albert A, et al. Urinary Biomarkers may Complement the Cleveland Score for Prediction of Adverse Kidney Events After Cardiac Surgery: A Pilot Study. Ann Lab Med. 2020;40(2):131-141.
23. Liu S, Che M, Xue S, et al. Urinary L-FABP and its combination with urinary NGAL in early diagnosis of acute kidney injury after cardiac surgery in adult patients. Biomarkers. 2013;18(1):95-101.
24. Piedrafita A, Siwy J, Klein J, et al. A universal predictive and mechanistic urinary peptide signature in acute kidney injury [published correction appears in Crit Care. 2022 Dec 29;26(1):406]. Crit Care. 2022;26(1):344.
25. Neyra JA, Hu MC, Minhajuddin A, et al. Kidney Tubular Damage and Functional Biomarkers in Acute Kidney Injury Following Cardiac Surgery. Kidney Int Rep. 2019;4(8):1131-1142.
26. Schley G, Köberle C, Manuilova E, et al. Comparison of Plasma and Urine Biomarker Performance in Acute Kidney Injury. PLoS One. 2015;10(12):e0145042.
27. Levante C, Ferrari F, Manenti C, et al. Routine adoption of TIMP2 and IGFBP7 biomarkers in cardiac surgery for early identification of acute kidney injury. Int J Artif Organs. 2017;40(12):714-718.
28. Choi N, Rigatto C, Zappitelli M, et al. Urinary Hepcidin-25 Is Elevated in Patients That Avoid Acute Kidney Injury Following Cardiac Surgery. Can J Kidney Health Dis. 2018;5:2054358117744224.
29. Haase M, Bellomo R, Albert C, et al. The identification of three novel biomarkers of major adverse kidney events. Biomark Med. 2014;8(10):1207-1217.
30. Oezkur M, Magyar A, Thomas P, et al. TIMP-2*IGFBP7 (Nephrocheck®) Measurements at Intensive Care Unit Admission After Cardiac Surgery are Predictive for Acute Kidney Injury Within 48 Hours. Kidney Blood Press Res. Published online July 27, 2017.
31. Ho J, Reslerova M, Gali B, et al. Urinary hepcidin-25 and risk of acute kidney injury following cardiopulmonary bypass. Clin J Am Soc Nephrol. 2011;6(10):2340-2346.
32. Ko SW, Chi NH, Wu CH, et al. Hemojuvelin Predicts Acute Kidney Injury and Poor Outcomes Following Cardiac Surgery. Sci Rep. 2018;8(1):1938.
33. Wang JJ, Chi NH, Huang TM, et al. Urinary biomarkers predict advanced acute kidney injury after cardiovascular surgery. Crit Care. 2018;22(1):108.
34. Moriyama T, Hagihara S, Shiramomo T, Nagaoka M, Iwakawa S, Kanmura Y. Comparison of three early biomarkers for acute kidney injury after cardiac surgery under cardiopulmonary bypass. J Intensive Care. 2016;4:41.
35. Merchant ML, Brier ME, Slaughter MS, Klein JB, McLeish KR. Biomarker enhanced risk prediction for development of AKI after cardiac surgery. BMC Nephrol. 2018;19(1):102.
36. Bernardi MH, Wagner L, Ryz S, et al. Urinary neprilysin for early detection of acute kidney injury after cardiac surgery: A prospective observational study. Eur J Anaesthesiol. 2021;38(1):13-21.
37. Miller D, Eagle-Hemming B, Sheikh S, et al. Urinary extracellular vesicles and micro-RNA as markers of acute kidney injury after cardiac surgery. Sci Rep. 2022;12(1):10402.
38. Arvin P, Samimagham HR, Montazerghaem H, Khayatian M, Mahboobi H, Ghadiri Soufi F. Early detection of cardiac surgery‑associated acute kidney injury by microRNA-21. Bratisl Lek Listy. 2017;118(10):626-631.
39. Wu X, Qiu F, Jin X, Zhou J, Zang W. ATF3: a novel biomarker for the diagnosis of acute kidney injury after cardiac surgery. Ann Transl Med. 2021;9(22):1655.
40. Gardner DS, Allen JC, Goodson D, et al. Urinary Trace Elements Are Biomarkers for Early Detection of Acute Kidney Injury. Kidney Int Rep. 2022;7(7):1524-1538.
41. Brown JR, Thiessen-Philbrook H, Goodrich CA, et al. Are Urinary Biomarkers Better Than Acute Kidney Injury Duration for Predicting Readmission?. Ann Thorac Surg. 2019;107(6):1699-1705.
42. Lakhal K, Bigot-Corbel E, Sacchetto E, et al. Early recognition of cardiac surgery-associated acute kidney injury: lack of added value of TIMP2 IGFBP7 over short-term changes in creatinine (an observational pilot study). BMC Anesthesiol. 2021;21(1):244.
43. Zaouter C, Potvin J, Bats ML, Beauvieux MC, Remy A, Ouattara A. A combined approach for the early recognition of acute kidney injury after adult cardiac surgery. Anaesth Crit Care Pain Med. 2018;37(4):335-341.
44. Finge T, Bertran S, Roger C, et al. Interest of Urinary [TIMP-2] × [IGFBP-7] for Predicting the Occurrence of Acute Kidney Injury After Cardiac Surgery: A Gray Zone Approach. Anesth Analg. 2017;125(3):762-769.
45. Grieshaber P, Möller S, Arneth B, et al. Predicting Cardiac Surgery-Associated Acute Kidney Injury Using a Combination of Clinical Risk Scores and Urinary Biomarkers. Thorac Cardiovasc Surg. 2020;68(5):389-400.
46. Couturier C, Maillard N, Mariat C, et al. Prevention of cardiac surgery-associated acute kidney injury by risk stratification using (TIMP-2)*(IGFBP7). Biomark Med. 2021;15(14):1201-1210.
47. Wetz AJ, Richardt EM, Wand S, et al. Quantification of urinary TIMP-2 and IGFBP-7: an adequate diagnostic test to predict acute kidney injury after cardiac surgery?. Crit Care. 2015;19(1):3.
48. Vandenberghe W, Van Laethem L, Herck I, et al. Prediction of cardiac surgery associated - acute kidney injury (CSA-AKI) by healthcare professionals and urine cell cycle arrest AKI biomarkers [TIMP-2]*[IGFBP7]: A single center prospective study (the PREDICTAKI trial). J Crit Care. 2022;67:108-117.
49. Esmeijer K, Schoe A, Ruhaak LR, et al. The predictive value of TIMP-2 and IGFBP7 for kidney failure and 30-day mortality after elective cardiac surgery. Sci Rep. 2021;11(1):1071.
50. Pilarczyk K, Edayadiyil-Dudasova M, Wendt D, et al. Urinary [TIMP-2]*[IGFBP7] for early prediction of acute kidney injury after coronary artery bypass surgery. Ann Intensive Care. 2015;5(1):50.
51. Alam A, Jamil AK, Van Zyl JS, et al. Urinary Cell-Cycle Arrest Biomarkers as Early Predictors of Acute Kidney Injury After Ventricular Assist Device Implantation or Cardiac Transplantation. J Cardiothorac Vasc Anesth. 2022;36(8 Pt A):2303-2312.
52. Engelman DT, Crisafi C, Germain M, et al. Stress Biomarkers Do Not Correlate With Risk Factors for Kidney Injury After Cardiac Surgery. Ann Thorac Surg. 2021;112(2):532-538.
53. Meersch M, Schmidt C, Van Aken H, et al. Urinary TIMP-2 and IGFBP7 as early biomarkers of acute kidney injury and renal recovery following cardiac surgery. PLoS One. 2014;9(3):e93460.
54. Wang Y, Zou Z, Jin J, et al. Urinary TIMP-2 and IGFBP7 for the prediction of acute kidney injury following cardiac surgery. BMC Nephrol. 2017;18(1):177.
55. Mayer T, Bolliger D, Scholz M, et al. Urine Biomarkers of Tubular Renal Cell Damage for the Prediction of Acute Kidney Injury After Cardiac Surgery-A Pilot Study. J Cardiothorac Vasc Anesth. 2017;31(6):2072-2079.
56. Irqsusi M, Beckers J, Wiesmann T, et al. Urinary TIMP-2 and IGFBP-7 protein levels as early predictors of acute kidney injury after cardiac surgery. J Card Surg. 2022;37(4):717-724.
57. Yu PJ, Rodriguez G, Cassiere H, et al. Use of TIMP-2 and IGFBP-7 for prediction of postoperative acute kidney injury after cardiac surgery. Clin Nephrol. 2022;98(6):288-295.
