# Supplementary material for: Combination of urinary biomarkers can predict cardiac surgery-associated acute kidney injury: a systematic review and meta-analysis
Source: Ann Intensive Care. 2025 Mar 29;15:45. doi: 10.1186/s13613-025-01459-7 (PMC11953499; doi:10.1186/s13613-025-01459-7)

**SUPPLEMENTARY TABLES**

Supplementary Table 1. Urinary biomarker measurements in included studies

| **Author, year, country** | **Urinary Biomarker** | **AKI**  **definition** | **Number of patients with AKI** | **Target** | **Urinary biomarker measurement time-points** |
| --- | --- | --- | --- | --- | --- |
| Cummings et al 2019, USA | TIMP2xIGFBP7 | KDIGO | 91 | Stage 2+3 AKI | induction, 30min on CPB, end of CPB, ICU admission, postoperative 6h, postoperative Day1,2,3 |
| Lakhal et al 2021, France | TIMP2xIGFBP7 | KDIGO | 27 | All AKI | insertion of urin cath, end of CPB, 6h post CPB. postoperative Day1 |
| Zaouter, 2018, France | TIMP2xIGFBP7 | KDIGO | 37 | All AKI | postoperative 12h |
| Finge, 2017, France | TIMP2xIGFBP7 | KDIGO | 34 | All AKI | postoperative 3h |
| Grieshaber, 2019, Germany | TIMP2xIGFBP7 | KDIGO | 40 | All AKI, Stage 2+3, Stage 3 | postoperative 4h |
| Couturier, 2021, France | TIMP2xIGFBP7 | KDIGO | 38 | All AKI | postoperative 4h |
| Wetz, 2015, Germany | TIMP2xIGFBP7 | KDIGO | 16 | All AKI | postoperative 0h, postoperative day 1, induction, 4h post CPB |
| Vandenberghe, 2022, Netherlands | TIMP2xIGFBP7 | KDIGO | 75 | All AKI | 4h postoperative |
| Esmeijer, 2021, Netherlands | TIMP2xIGFBP7 | KDIGO | 22 | RRT | preoperative, ICU admission |
| Pilarczyk, 2015, Germany | TIMP2xIGFBP7 | KDIGO | 19 | Stage 2+3 AKI | postoperative 4h, postoperative day 1, max in postoperative 24h |
| Piedrafita, 2022, France | TIMP2xIGFBP7, NGAL | KDIGO | 175 | All AKI, Stage 2+3 AKI | postoperative 4h |
| Alam, 2022, USA | TIMP2xIGFBP7 | KDIGO | 48 | All AKI, Stage 2+3 AKI | postoperative 6h |
| Engelman, 2021, USA | TIMP2xIGFBP7 | KDIGO | 42 | All AKI | postoperative day1 |
| Meersch, 2014, Germany | TIMP2xIGFBP7 | KDIGO | 26 | All AKI | 4h post CPB, 12 h post CPB, 24h post CPB, max in postoperative 24h |
| Yimei Wang, 2017, China | TIMP2xIGFBP7 | KDIGO | 20 | All AKI, Stage 2+3 | postoperative 4h |
| Mayer, 2017, Switzerland | TIMP2xIGFBP7 | KDIGO | 9 | All AKI | 1h post start CPB, 4h post CPB |
| Oezkur, 2017, Germany | TIMP2xIGFBP7 | KDIGO | 35 | All AKI | preoperative, ICU admission, 24h postoperative |
| Irqsusi, 2022, Germany | TIMP2xIGFBP7 | KDIGO | 14 | All AKI | 0h postoperative, 24h postoperative |
| Yu, 2022, USA | TIMP2xIGFBP7 | KDIGO | 19 | All AKI | 0h post CPB. postoperative Day1, max in postoperative 24h |
| Moriyama, 2016, Japan | NGAL, L-FABP, angiotensinogen | KDIGO | 11 | All AKI | postoperative 3h |
| DeLoor, 2017, Belgium | NGAL,UCHI3L1 | KDIGO | 108 | All AKI, AKI Stage 2 | ICU admission |
| Lee1, 2019, Taiwan | NGAL | KDIGO | 92 | All AKI, RRT | postoperative 0h |
| Jie Hu, 2021, China | NGAL, NAG, KIM-1 | KDIGO | 71 | All AKI | end of CPB |
| Sheng-Wen Ko, 2018, Taiwan | NGAL, HJV | KDIGO | 51 | All AKI, Stage 2-3 | ICU admission, 3h postoperative, 6h postoperative, 12h postoperative, 24h postoperative |
| Liebetrau, 2013, Germany | NGAL | KDIGO | 47 | Stage 2+3 AKI | 4h postoperative |
| McIlroy, 2015, USA | NGAL | KDIGO | 155 | All AKI | induction, 1h post CPB, 3h post CPB, 18-24h post CPB |
| Fanning, 2016, New Zealand | NGAL | KDIGO | 31 | All AKI | 4h post CPB. 24h post CPB |
| Metzger, 2016, Germany | NGAL, KIM-1 | AKIN | 59 | All AKI | postoperative 12-18h |
| Liu, 2012, China | NGAL, L-FABP | AKIN | 26 | All AKI, Stage 2-3 | ICU admission, 2h post ICU admission |
| Matsui, 2012, Japan | NGAL, NAG, L-FABP | AKIN | 48 | All AKI | preoperative, postoperative 0h, postoperative 3h, postoperative 6h, postoperative 18h, postoperative 24h postoperative 48h |
| Sargentini, 2012, Italy | NGAL | AKIN | 15 | All AKI | 4h post CPB, 24h postCPB |
| Varela, 2015, Brazil | NGAL | AKIN | 16 | All AKI | 1h postoperative, 6h postoperative, 24h postoperative |
| Wagener, 2006, USA | NGAL | RIFLE | 16 | All AKI | 0h postoperative, 1h postoperative, 3h postoperative, 18h postoperative, 24h postoperative, peak |
| Prowle, 2015, Australia | NGAL, L-FABP, alpha-GST, pi-GST, hepcidin | RIFLE | 25 | All AKI, RIFLE-I, RRT/death | ICU admission, 24h postoperative |
| JianJhongWang, 2018, Taiwan | NGAL, KIM-1, HJV, alpha-GST, pi-GST | KDIGO | 46 | AKI Stage 2-3 | postoperative 3,6,12,24h |
| Koyner, 2010, USA | NGAL, KIM-1, Cystatin-C, alpha-GST, pi-GST | AKIN | 46 | AKI Stage 1, Stage 3 | preoperative, postoperative 0, 6h, early max |
| Parikh, 2011, USA | NGAL, IL-18 | AKIN | 60( stage 2+3) | Stage 2+3, RRT | 6h postoperative |
| Schley, 2015, Germany | NGAL, L-FABP, KIM-1, Cystatin-C, alpha-1-microglobulin, alpha-GST | AKIN | 37 | All AKI | preoperative, 2h post ICU admission, postoperative 4h, postoperative 24h |
| Miaolin Che, 2010, China | NGAL, NAG, IL-18 | AKIN | 14 | All AKI | ICU admission, postoperative 2, 4, 10h |
| Liangos, 2009, USA | NGAL, NAG, KIM-1, IL-18, Cystatin-C, alpha-1-microglobulin | AKIN | 13 | All AKI | 2h postCPB |
| Paarman, 2013, Germany | NGAL, KIM-1, L-FABP | AKIN | 29 | All AKI | preoperative, postoperative 0h, postoperative 6h |
| Han, 2009, USA | NGAL, NAG, KIM-1 | AKIN | 36 | All AKI, early AKI | preoperative, end of CPB, 3h post CPB, 18h post CPB, 24h post CPB |
| Albert, 2020, Germany | NGAL, hepcidin, midkine, alpha-1-microglobulin | RIFLE | 9 | All AKI | 6h post start of CPB |
| Heise, 2011, Germany | NGAL, Cystatin-C, alpha-1-microglobulin | AKIN | 38 | All AKI | 6-18h postoperative |
| Tidbury, 2019, UK, post hoc of RCT | NGAL | KDIGO | 54 | All AKI | preoperative, 6h postoperative, 24h postoperative, 48h postoperative |
| Elmedany, 2017, Egypt, prosp obs | NGAL, KIM-1 | AKIN | 11 | All AKI | induction, 2h postCPB, 6hpostCPB, 12h postCPB, 24h post CPB |
| Averdunk, 2019, Germany, prosp obs | NGAL, SLP1 | KDIGO | 22 | All AKI | 0h postoperative |
| Sun, 2021, China, prosp obs | NGAL, KIM-1, galectin | KDIGO | 17 | All AKI | 0h postoperative |
| Haase2, 2014, Germany, nested cohort of RCT | NGAL | RIFLE | 23 | All AKI | preoperative, ICU admission, 24h postoperative |
| Garcia-Alvarez, 2015, Spain, prosp observ | NGAL | KDIGO | 104 | All AKI, Stage 2 and 3 | ICU admission, 24h postoperative, 48h postoperative |
| Quian, 2019, China, prosp obs | NGAL, klotho | AKIN | 33 | All AKI | 0h postoperative, 2h post ICU admission, 4h postoperative |
| Xin, 2008, China, prosp obs | NGAL, IL-18 | Se Creat>50% | 9 | All AKI | 2h postoperative |
| Kar, 2021, Bangladesh, prosp obs | NGAL | AKIN | 8 | All AKI | nd |
| Wagener2, 2008, USA, prosp obs | NGAL | AKIN | 85 | All AKI | 0h, 3h, 18h, 24h postoperative |
| Wan, 2008, China, prosp obs | NGAL | Se Creat>50% | 9 | All AKI | 2h postoperative |
| Munir, 2013, Pakistan, prosp obs | NGAL | AKIN | 11 | All AKI | 4h postoperative |
| Mori, 2014, Japan, prosp obs | LFABP | AKIN | 22 | All AKI | end of DHCA |
| TaoHanLee, 2021, Taiwan, prosp obs | LFABP | KDIGO | 59 | All AKI | 4-6h postoperative, 16-18h postoperative |
| Parikh2, 2013, USA, prosp obs | LFABP, KIM-1 | RRT or doubling of Se creat (AKIN) | 60 (stage 2-3) | Stage 2-3 | 6h, 12h postoperative |
| Katagiri, 2012, Japan, prosp obs | LFABP,NAG | AKIN | 28 | All AKI | preoperative, postoperative 0h, 4h, 12h |
| McIlroy, 2018, USA, prosp obs | KIM-1, IL-18, CysC | KDIGO | 155 | All AKI | induction, 1h post CPB, 3h post CPB, postoperative D1 |
| Khreba, 2019, Egypt, prosp obs | KIM-1 | KDIGO | 27 | All AKI | postoperative 3h |
| Silverton, 2021, USA, prosp obs | KIM-1, IL-18 | KDIGO | 22 | All AKI | end of surgery, 12h post CPB |
| Koyner, 2013, USA, prosp obs | CysC | AKIN | 472 | Stage 1, Stage 2+3 | postoperative 6h, postoperative 12h |
| Susantitaphong, 2013, USA, prosp obs | alpha-GST, pi-GST | AKIN | 72 | All AKI, Stage 2-3 | preoperative, 2h postoperative |
| Shu, 2016, Taiwan, prosp obs | alpha-GST, pi-GST | KDIGO | 38 | Stage 2-3 | 3h, 6h, 9h,12h,24h postoperative |
| Yavuz, 2009, Turkey, prosp obs | alpha-GST | RIFLE(creatinine) | 16 | RIFLE F | preoperative, 24h postoperative |
| Neyra, 2019, | SeCysC, NGAL, KIM-1 | KDIGO | 23 | All AKI | preoperative, 6h post CPB, D1,D2,D3,D4 |
| Levante, 2017 | TIMP2xIGFBP7 | KDIGO | 10 | All AKI | Not specified |
| Choi, 2018 | hepcidin | KDIGO | 41 | All AKI | Baseline, start of CPB, 1h onCPB, ICU admission, Postoperative D1, postoperative D3-5 |
| Ho, 2011, Canada, prosp obs | hepcidin | Se creat >50% | 28 | All AKI | postoperative D1 |
| ChanganWang, 2017, China, prosp obs | IL-18 | Se creat>50% | 22 | All AKI | 2h postCPB |
| Merchant, 2018, USA | angiotensinogen | KDIGO | 21 | All AKI | 4h preoperative |
| Prowle, 2012, Australia, RCT | hepcidin | RIFLE (creat) | 25 | All AKI, RIFLE I, RIFLE-F | preoperative, ICU admission, 24h postoperative |
| Kambhampati,2013, USA | IL-18 | AKIN | 27 | All AKI | 24h post start of surgery |
| Haase, 2008, Australia | IL-18 | Se Creat rise>50% | 20 | All AKI | ICU admission, 24h post CPB |
| Kanchi, 2023, India | TIMP2xIGFBP7 | KDIGO | 13 | All AKI | 4h postoperative |
| Monaco, 2024, multinational | TIMP2xIGFBP7 | KDIGO | 38 | All AKI, Stage 2+3 | 4h postCPB |
| Lacquaniti, 2023, Italy | TIMP2xIGFBP7 | KDIGO | 117 | All AKI | 4h post ICU admission |
| Yun, 2024, Italy | TIMP2xIGFBP7 | KDIGO | 134 | All AKI | 6-12h postoperative |
| Ghaheh, 2021, Iran | KIM-1, NGAL | Se Creat rise>50% | 45 | All AKI | Postoperative D1 |
| Chica, 2024, Switzerland | NAG, NGAL | AKIN | 19 | All AKI, Stage 2-3 | ICU admission |
| Fang, 2022, China | NAG | KDIGO | 214 | All AKI | 0h postoperative |
| Takaki, 2024, Japan | NAG | KDIGO | 32 | All AKI | ICU admission |
| Udzik, 2022, Poland | NGAL | KDIGO | 87 | All AKI | 6h postoperative |

Supplementary table 2. Risk of bias assessment by QUADAS-2


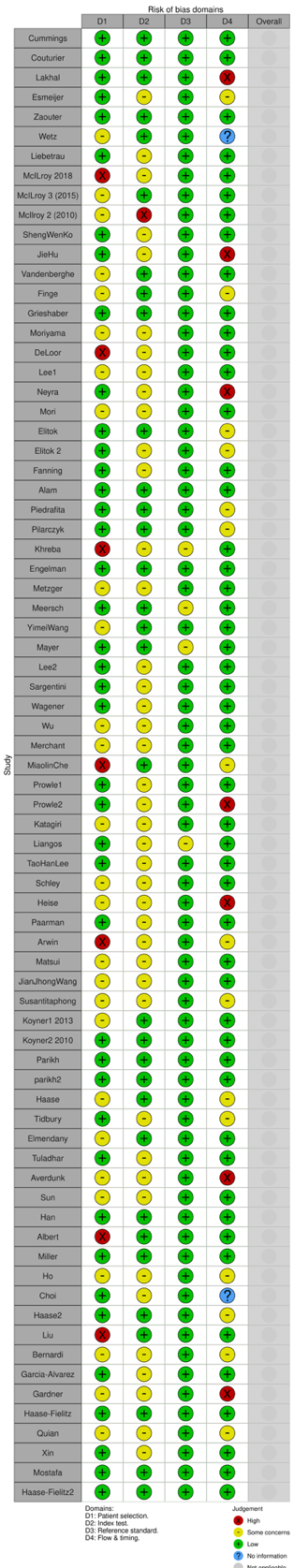


Supplementary Table 3

GradePro Assessment


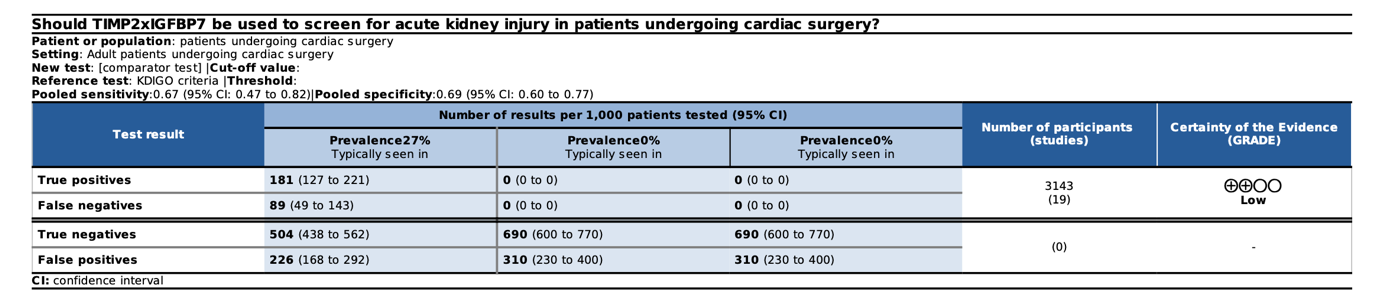

Supplement: Supplementary file 2 — Supplementary material 2. [file 13613_2025_1459_MOESM2_ESM.docx]
